# Effects of different plasma target concentrations of remifentanil on the MAC_BAR_ of sevoflurane in children with laparoscopic surgery

**DOI:** 10.1186/s12871-021-01453-z

**Published:** 2021-09-24

**Authors:** Dan Wang, Juan Xu, Xiao-Lin Yang, Yan-Xia Guo, Ping-Ping Jiang, Guo-Yuan Zhang

**Affiliations:** 1 Present Address: Department of Anaesthesia, Affiliated Hospital of North Sichuan Medical College, Nanchong, 637000 Sichuan China; 2 Present Address: Department of Clinical Laboratory, Affiliated Hospital of North Sichuan Medical College, Nanchong, 637000 Sichuan China

**Keywords:** Remifentanil, Sevoflurane, Children, Pneumoperitoneum stimulus, Minimum alveolar concentration (MAC)

## Abstract

**Background:**

To investigate the effects of different plasma target concentrations of remifentanil on the minimum alveolar concentration (MAC) for blocking adrenergic response (BAR) of sevoflurane in children with laparoscopic herniorrhaphy.

**Methods:**

Seventy-five children with 3-7 years old scheduled for laparoscopic herniorrhaphy were randomly divided into group R_0_, group R_1,_ and group R_2_ according to different remifentanil plasma target concentration (0, 1, and 2 ngml^-1^), respectively. The MAC_BAR_ of sevoflurane was determined by the up-and-down and sequential method in each group. The concentrations of epinephrine and noradrenaline were also determined at corresponding time points.

**Results:**

A total of 52 child patients were used among the anticipated 75 patients. In groups R_0_, R_1,_ and R_2_, the MAC_BAR_ of sevoflurane was (3.29 ± 0.17) %, (2.12 ± 0.10) % and (1.29 ± 0.11) %, respectively, and a significant difference was found among the three groups (*P*<0.05). The changes of epinephrine and noradrenaline concentrations in each group before and after insufflation of carbon dioxide pneumoperitoneum showed no significant differences.

**Conclusion:**

Remifentanil by target-controlled infusion can effectively reduce the MAC_BAR_ of sevoflurane during laparoscopic surgery in children. At a similar effect of MAC_BAR_, both the changes of epinephrine and noradrenaline concentrations are not affected by the infusion of different remifentanil target concentrations.

**Trial registration:**

The trial was registered at http://www.chictr.org.cn(ChiCTR1800019393, 8, Nov, 2018).

## Background

Laparoscopic surgery has been widely used in pediatric surgery in recent years. Compared with the traditional surgery method, it possesses many advantages, such as slight trauma, rapid postoperative recovery, low incidence of infection and less pain, etc. [1]. Due to the complicated effect of carbon dioxide (CO_2_) pneumoperitoneum stimulus on children's hemodynamics [2], it raises a higher requirement for anesthesiologists to use drugs reasonably and maintain hemodynamic stability skillfully. Previous studies have shown that anesthesia with sevoflurane alone requires a higher minimum alveolar concentration (MAC) to block adrenergic response (BAR) in adult patients with CO_2_ pneumoperitoneum stimulus [3, 4]. However, a high concentration of sevoflurane for only use is usually associated with hemodynamic instability, myocardial depression, and postoperative delirium in children [5]. Nowadays, balanced anesthesia is used more frequently to provide better pain control and also to achieve an adequate depth of anesthesia. It is often necessary to use other analgesics to reduce sevoflurane’s concentration and its side effects. Remifentanil is a strong short-acting opioid, does not rely on liver and kidney metabolism, and is suitable for target-controlled infusion. Therefore, this study aims to investigate the effects of different remifentanil plasma target concentrations on the MAC_BAR_ of sevoflurane in children with laparoscopic surgery.

## Methods

### Subjects

This study was approved by the Ethics Committee of the Affiliated Hospital of North Sichuan Medical College, Nanchong, China (Approved No. 2018ERR009). Written informed consent was obtained from each child’s guardian. All experiment procedures (consisted of invasive manipulation) and data collection were conducted with prior informed consents. The manuscript adheres to CONSORT guidelines and was registered with the Chinese Clinical Trials Registry at http://www.chictr.org.cn (ChiCTR1800019393, principal investigator: Juan XU, date of registration: Nov. 8, 2018).

**Fig. 1:**
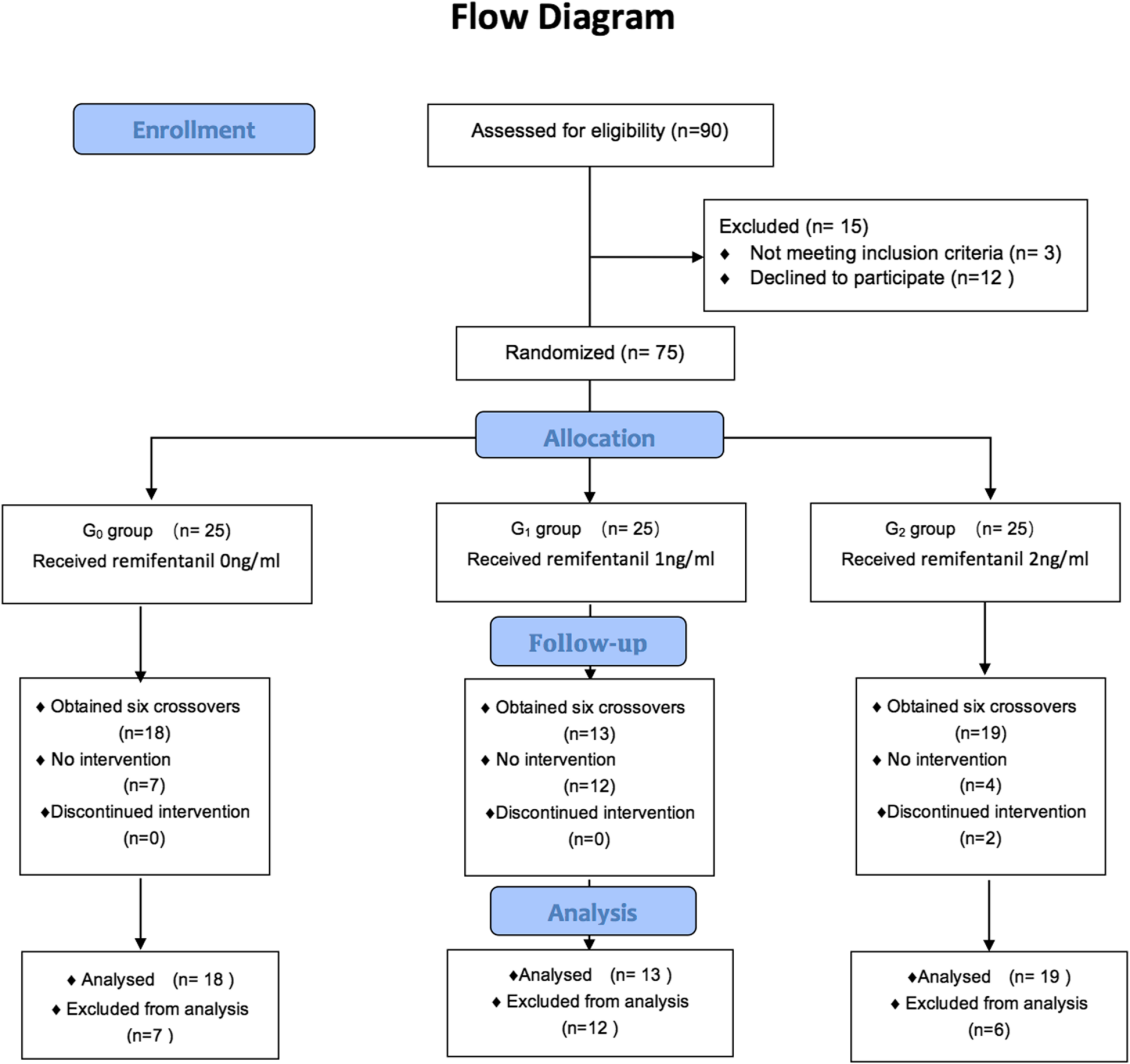
Flowchart of the patient selection and analysis process for the study. In this study,75 patients were randomly allocated into 3 groups with 25 patients in each group. To obtain six crossover points of positive vs. negative response in each group, 18, 13 and 19 (not shown the 2 cases for discontinue intervention due to hypotension) patients in groups R_0_, R_1_ and R_2_ were needed respectively. Finally, remaining 23 patients did not undergo the experimental intervention

This research was conducted between November 2018 and June 2019. Seventy-five child patients, scheduled for laparoscopic herniorrhaphy, with American Society of Anesthesiologists (ASA) physical status I-II, aged between 3 and 7 years, were selected. Exclusion criteria included a history of cardiovascular, brain, liver, kidney, or hematological disease; a history of allergies to inhalation anesthetics or opioids; a history of recent upper respiratory tract infection. The flowchart of patients through the trial is shown in Fig. 1.

### Study design

All children were randomly assigned to three groups (R_0_, R_1_, R_2_) with 25 cases in each group according to computer-generated randomization. Children in the three groups were anaesthetized by inhalation of sevoflurane and intravenous infusion of remifentanil with different plasma target concentrations (0, 1, 2, ng ml^-1^), respectively. During the creation of CO_2_ pneumoperitoneum, the sympathetic adrenergic response was monitored in all patients. A positive response was defined as an increased heart rate (HR) or mean arterial pressure (MAP) over its baseline value more than 20%. On the contrary, if the increase of HR and MAP was less than 20% of its baseline value, the sympathetic adrenergic response was defined as a negative response. The mean value of MAP or HR measured 3 and 1 min before pneumoperitoneum stimulus was defined as its baseline value. The mean value of HR or MAP measured 1 and 3 min after the pneumoperitoneum pressure maintained stable was defined as its changed value. Patients would be excluded from the experiment if hypotension or bradycardia occurred at the period of determination. Hypotension was defined as systolic blood pressure (SBP) (5th percentile at 50th height percentile), less than 2 x age in years + 65 and was treated with intravenous ephedrine. Similarly, bradycardia was defined as HR < 80 bpm and was treated with intravenous atropine [6, 7].

### Anesthesia administration

#### Induction

All children were fasted for 6 h and not allowed to drink water for 2 h before operation, and not received premedication routinely. Before induction of anesthesia, a venous channel was established and infused with compound sodium chloride solution at a rate of 10 mlˑkg^-1^ˑh^-1^. Electrocardiogram, pulse oxygen saturation, non-invasive blood pressure were routinely monitored with a PM-9000 express monitor (Mindray Medical International Limited, Shenzhen, China), and depth of anesthesia was monitored by using bispectral index (BIS) (Canwell Medical International Limited, Zhejiang, China). In each group, anesthesia was induced by inhalation of 7% sevoflurane with 100% oxygen. After children had lost their consciousness, the inhaled sevoflurane concentration was reduced appropriately, and 1 μgkg^-1^ remifentanil and 0.6 mgkg^-1^ rocuronium were intravenously injected. After tracheal intubation, sevoflurane concentration was adjusted to a preset end-tidal concentration of 3.0%, 2.2% and 1.4% in group R_0_, group R_1_ and group R_2_, respectively. Sevoflurane concentration was monitored by a multifunctional monitor (Shenzhen Mindray Biomedical Co., Ltd., PM9000). At the same time, remifentanil was administered by target-controlled infusion in each group with the Minto model using a micro pump (TCI-I, ver 4.0, Guangxi VERYARK Technology Co., Ltd). The degree of neuromuscular relaxation (2 Hz for 1.5 s every 11.5 s) was continuously assessed by acceleromyography using a TOF-watch SX system (Veryark-TOF, Guangxi, China), starting when the children were unconscious [8, 9].

#### Measurement of MAC_BAR_

When the preset end-tidal sevoflurane concentration had maintained stable at least 20 minutes, CO_2_ pneumoperitoneum was established, and its pressure was set at 9 mmHg with a flow rate of 2 Lmin^-1^. The first child’s preset end-tidal sevoflurane concentration in each group was obtained from our preliminary test. The next child’s end-tidal sevoflurane concentration for maintenance in each group would be adjusted based on the result of the previous child’s cardiovascular response. If the response was positive (negative), the subsequent child’s end-tidal sevoflurane concentration would be increased (decreased) by 0.2%. The person for recording the data was blinded to the plasma target-controlled remifentanil concentrations used in all groups.

The test was over in each group when six crossing points of a positive versus negative response or a negative versus positive response in the pre and the next child had occurred. The MAC_BAR_ of sevoflurane in each group was calculated as the mean value of the end-tidal sevoflurane concentrations corresponding to the six crossing points. After the above test had been done, 0.1 mg kg^-1^ of midazolam was given intravenously to prevent a potential intraoperative awareness. All the children were received a routine intravenous and inhaled combined anesthesia. The BIS value was maintained between 40 and 60. The administration of sevoflurane and remifentanil were discontinued 5 minutes before the end of operation, and 1.5 μgkg^-1^ of fentanyl was intravenously injected for analgesia. All children were transferred to the pediatric intensive care unit.

#### Determination of blood samples

Arterial blood samples (each for 3 ml) were collected 3 min before and after CO_2_ pneumoperitoneum with sodium-heparin-containing tubes. Soon after, the plasma was separated and frozen at -70°C in a refrigerator until analysis. After the sample collection had been completed, the concentrations of epinephrine (E) and norepinephrine (NE) were measured using a method that has been described previously [3].

#### Statistical analysis

Statistical analysis was performed using SPSS22.0 software. All measurement data were expressed as mean ± SD. Only these data from the 12 cases at 6 crossing points of a positive (negative) versus negative (positive) response in each group were analyzed. The delta HR, delta MAP, delta E, and delta NE value was calculated as the difference between its change value and baseline value, respectively. One-way analysis of variance (ANOVA) for the complete random design was used to compare the differences of age, weight, MAC_BAR_, delta HR, delta MAP, delta E, and delta NE among the three groups, respectively. The sex constituent ratio was tested by Fisher's exact probability among the three groups. *P* value <0.05 was considered as a statistical significance.

## Results

**Fig. 2:**
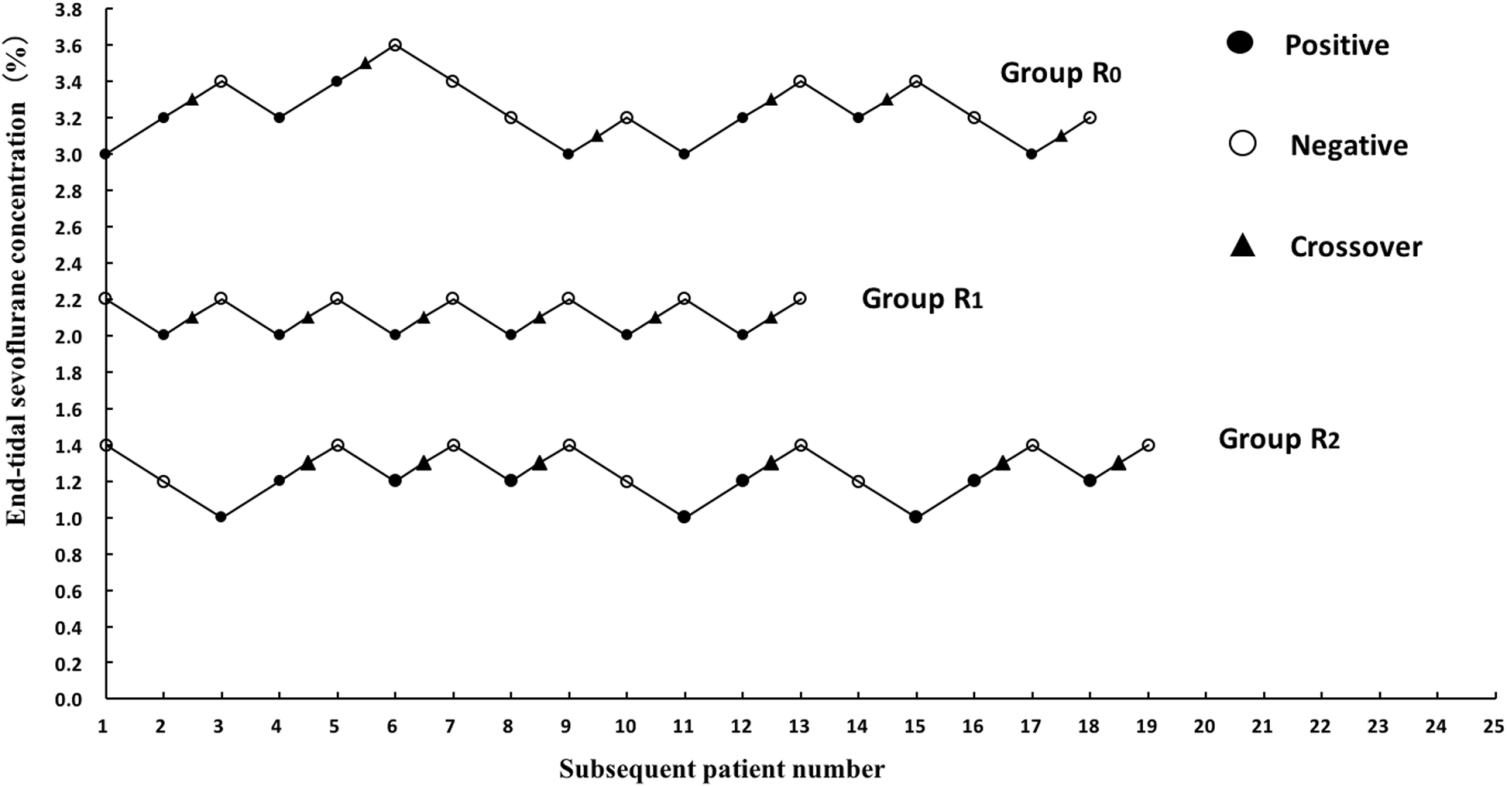
The measurement of MAC_BAR_ of sevofluane in 3 groups. The plasma target concentration of remifentanil in groups R_0_, R_1_ and R_2_ was 0, 1 and 2 ng ml^-1^, respectively. An empty (solid) circle represents a negative (positive) reaction to hemodynamic parameters, and a triangle indicates an intersection of a negative reaction with a positive reaction. To get six crossovers, 18, 13 and 19 patients (not shown the 2 cases for withdrawn) were needed in groups R_0_, R_1_ and R_2_, respectively

A total of 52 cases from the anticipant 75 child patients were used in this study. Two cases in group R_2_ due to hypotension were withdrawn from the study. Ultimately, to obtain six crossing points (Fig. 2), 18, 13, and 21 cases were used in groups R_0_, R_1,_ and R_2_, respectively. The general information and the MAC_BAR_ of sevoflurane in the three groups were shown in Table 1. Target-controlled infusion of 1 ngml^-1^ and 2 ngml^-1^ remifentanil can reduce the MAC_BAR_ of sevoflurane in children by 36% and 61%, respectively (*P*<0.05). The baseline values of HR and MAP in groups R_1_ and R_2_ were lower than those in group R_0_ (*P*<0.05), but no significant differences were found between group R_1_ and group R_2_ (*P*> 0.05). No significant differences were found in the delta HR, delta MAP, delta E, and delta NE among the three groups, respectively (*P*>0.05).

**Table 1:** The comparison of patients’ characteristics, delta HR, delta MAP, delta E, and delta NE among three groups. The data of 2 cases that were withdrawn from group R_2_ were not included in this table. Values are presented as mean ± SD or n. The baseline value of each parameter was the average value measured 3 and 1 min before CO_2_ pneumoperitoneum. The value of delta represents the difference between before and after pneumoperitoneum stimulation. HR, heart rate; MAP, mean arterial pressure; E, epinephrine concentration; NE, norepinephrine concentration. ^#^*P* < 0.05, compared with group R_0_; ^*^ *P* < 0.05, compared with group R_1_

Parameter	Group R_**0**_	Group R_**1**_	Group R_**2**_	P values			
				R_0_&R_1_&R_2_	R_0_&R_1_	R_0_&R_2_	R_1_&R_2_
Age (years)	4.6 ± 1.0	5.5 ± 1.1	4.9 ± 1.0	0.147	0.055	0.553	0.156
Body weight (kg)	17.1 ± 2.5	18.3 ± 2.2	17.5 ± 2.1	0.359	0.188	0.901	0.222
Male/Female (n)	16/2	12/1	16/3	0.912	0.802	0.968	0.833
MAC_BAR_ (%)	3.29 ± 0.17	2.12 ± 0.10^#^	1.29 ± 0.11^#*^	0.000	0.000	0.000	0.000
Baseline HR (bpm)	135 ± 13	106 ± 15 ^#^	94 ± 11 ^#^	0.000	0.000	0.000	0.026
Delta HR	9 ± 12	7 ± 13	8 ± 5	0.863	0.595	0.732	0.849
Baseline MAP (mmHg)	68 ± 5	59 ± 2 ^#^	54 ± 6 ^#^	0.000	0.000	0.000	0.013
Delta MAP	8 ± 6	11 ± 10	13 ± 9	0.344	0.362	0.151	0.589
Baseline E (ngml^-1^)	2.20 ± 0.63	2.10 ± 0.80	2.05 ± 0.91	0.900	0.764	0.655	0.883
Delta E	0.20 ± 1.23	0.33 ± 0.95	-0.06 ± 1.21	0.693	0.787	0.572	0.405
Baseline NE (pgml^-1^)	540.30 ± 65.64	498.40 ± 61.66	464.09 ± 76.91	0.373	0.453	0.164	0.510
Delta NE	65.31 ± 20.75	56.24 ± 21.01	52.85 ± 24.37	0.368	0.297	0.181	0.706

## Discussion

Previous studies have found that opioid analgesics can reduce the MAC_BAR_ or MAC of inhalation anaesthetic under skin-cutting stimulus both in children and adults [4, 10–12] and also can reduce the MAC_BAR_ of sevoflurane when using pneumoperitoneum stimulation in adults [3]. However, whether opioid analgesics have the same effect on sevoflurane’s MAC_BAR_ in children under pneumoperitoneum stimulus has not been reported. This study found that remifentanil plasma target concentrations 1 ngml^-1^ and 2 ngml^-1^ could make the MAC_BAR_ of sevoflurane (3.29%) in children decreased 36% and 61% under pneumoperitoneum stimulus, respectively. The decreased degree is very similar to our previous result when the same target concentrations of remifentanil were used in adult’s laparoscopic surgery (decreased 48% and 63%) [3]. It is also similar to another study’s result in adult patients with the similar plasma target concentrations of remifentanil by skin-cutting stimulus [13]. It means that a same plasma target concentration of remifentanil can induce a similar decreased degree of sevoflurane’s MAC_BAR_ no matter using pneumoperitoneum stimulus or incision stimulus either in adults or in children.

However, at a same target concentration of remifentanil, the MAC_BAR_ of sevoflurane in children using pneumoperitoneum stimulus is higher than that using skin incision stimulus, which may be mainly related to that pneumoperitoneum stimulus is more intensive than skin incision stimulus [3] because laparoscope pneumoperitoneum stimulus not only includes a direct stimulus of the needle to the skin but also include the stimulus of CO_2_ pneumoperitoneum pressure. A stronger pneumoperitoneum stimulus must induce a greater impact on children’s heart rate and blood pressure [14]. A deeper anaesthesia is required to inhibit an intense cardiovascular response. As all known, the setup of CO_2_ pneumoperitoneum will increase patient’s intra-abdominal pressure and thoracic pressure, which will induce a decrease of venous return and cardiac output and excite the sympathetic nervous system. In addition, the absorption of CO_2_ through the peritoneum can also indirectly stimulate the central nervous system and activate the sympathetic adrenal system [15]. As a result, it will lead to a significant increase in the secretion of cortisol, epinephrine, norepinephrine, rennin and vasopressin, and eventually manifest as an increase of patients’ blood pressure and heart rate [16]. However, when we analyzed the changes of plasma epinephrine and norepinephrine concentrations, heart rate, and blood pressure before and after pneumoperitoneum stimulus in pediatric patients with crossover points to cardiovascular response, no significant differences were found among the three groups, respectively (Table 1). It indicates that at a similar depth of anesthesia, the changes of plasma epinephrine and norepinephrine levels and hemodynamic parameters are consistent and not affected by different plasma target concentrations of remifentanil, which is consistent with the results of Zou's study in adult patients with laparoscopic surgery [3].

In this study, the HR and MAP in groups R_1_ and R_2_ were significantly lower than those in group R_0_ before the creation of pneumoperitoneum, which may be related to multiple mechanisms of remifentanil causing slow heart rate and low blood pressure, such as exciting vagus nerve, inhibiting sinus node’s self-regulation, and relaxing the peripheral vascular smooth muscle and so on [17, 18]. However, no severe hypotension and bradycardia occurred when 1 ngml^-1^of remifentanil target concentration was used. Only 2 cases experienced transient hypotension in the group with 2 ngml^-1^ of remifentanil target concentration. It can be quickly corrected by intravenous bolus injection of ephedrine 3-5mg. Our preliminary experiment found that severe hypotension or bradycardia would happen when the remifentanil plasma target concentration was more than 3 ngml^-1^, and the end-tidal sevoflurane concentration would be close to its MAC_awake_ value [19], which may appear intraoperative awareness. Therefore, we had not attempted to measure the MAC_BAR_ of sevoflurane using a higher remifentanil target concentration over 2 ngml^-1^.

Although the basal stress level and related plasma levels of stress hormones might be affected in children who were taken to the operating room without premedication. The premedication, like as midazolam and atropine, will also affect the changes of hemodynamics (such as HR and MAP). It may induce a dual effect with remifentanil on the MAC_BAR_ of sevoflurane, so that none of our patients received premedication in this study. During our study, as per the study protocol, the MAC_BAR_ of sevoflurane was calculated depending on the changes of MAP and HR values that were recorded after anesthesia induction and before the surgical incision and establishment of pneumoperitoneum. During this period, we maintained a stable hemodynamical status. Therefore, the measured result of sevoflurane MAC_BAR_ should be little affected by the stress situation before anaesthesia.

There are several limitations of our study. One of the limitations is that this study mainly focused on child patients at preschool age group between 3 and 7 years old. We did not choose infants, school-age and adolescent children in this study. The effects of infants, school-age and adolescent children on the MAC_BAR_ of sevoflurane with pneumoperitoneum stimulus needs further study. Another limitation is that we have not measured the actual plasma remifentanil concentration, although the Minto pharmacokinetic model for target-controlled infusion is safe in adults [20–23]. The accuracy of the Minto pharmacokinetic model of remifentanil in the pediatric population needs further study.

## Conclusions

Remifentanil by target-controlled infusion can significantly reduce the MAC_BAR_ of sevoflurane responding to laparoscopic pneumoperitoneum stimulus in children of 3 to 7 years old. In addition, at a similar effect of sevoflurane's MAC_BAR_, the changes of adrenergic response are similar.

## Data Availability

The datasets used and/or analyzed during the current study are available from the corresponding author on reasonable request. The corresponding author: Xiao-Lin Yang, Email: 879921874@qq.com.

## Abbreviations

MAC: Minimum alveolar concentration
MAC_BAR_: Minimum alveolar concentration for blocking adrenergic response
CO_2_: Carbon dioxide
ASA: American Society of Anesthesiologists
HR: Heart rate
MAP: Mean arterial pressure
SBP: Systolic blood pressure
BIS: Bispectral index
E: Epinephrine
NE: Norepinephrine
ANOVA: One-way analysis of variance
